# Supplementary material for: LINE1-Mediated Reverse Transcription and Genomic Integration of SARS-CoV-2 mRNA Detected in Virus-Infected but Not in Viral mRNA-Transfected Cells
Source: Viruses. 2023 Feb 25;15(3):629. doi: 10.3390/v15030629 (PMC10057545; doi:10.3390/v15030629)
Supplement: Supplementary file 1 [file viruses-15-00629-s001.zip › viruses-2203975-supplementary.pdf]

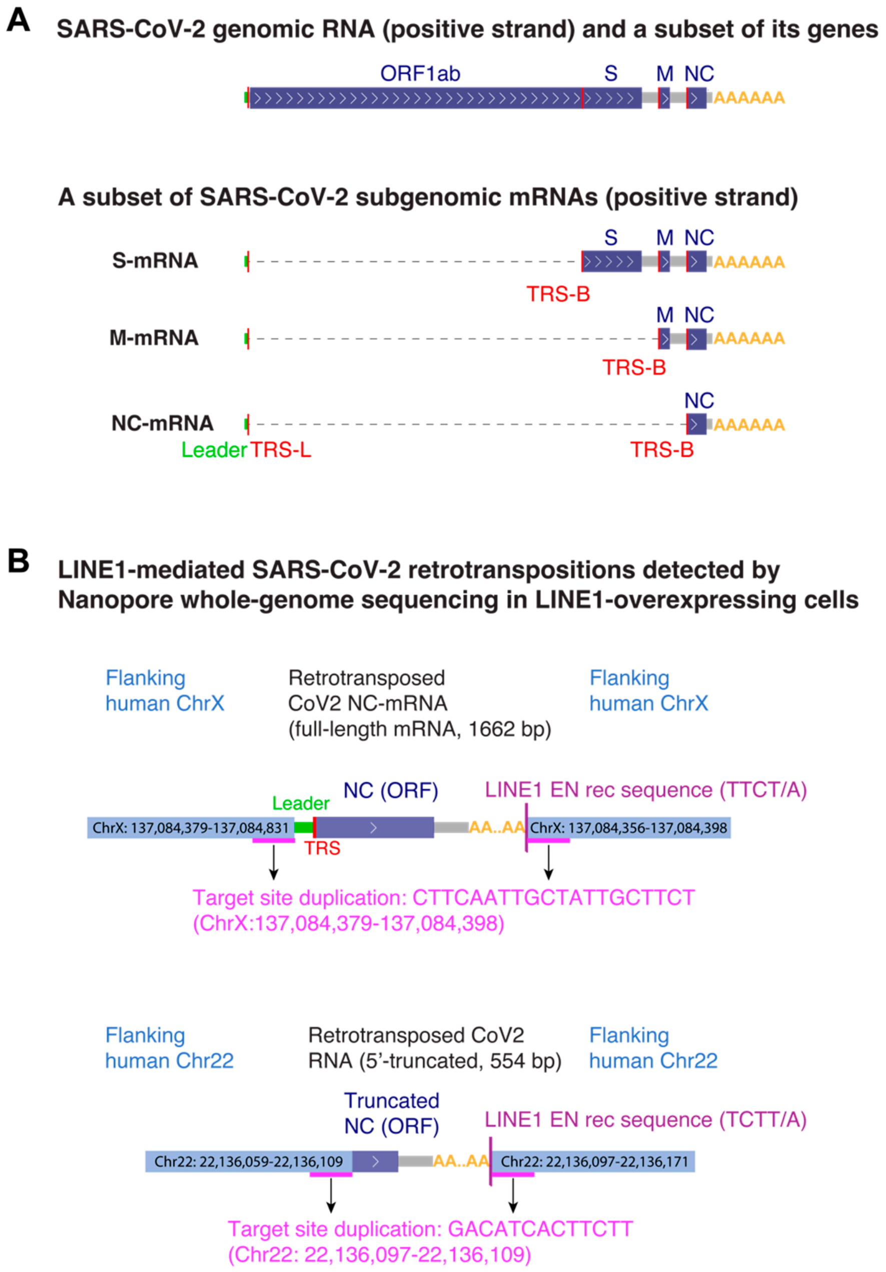

Supplementary Figure S1. SARS-CoV-2 RNA architecture and retrotransposition by a LINE1-mediated TPRT mechanism. A) Architectures of SARS-CoV-2 genomic RNA and a subset of subgenomic mRNAs with sequence features of 5'-leader (green), genes (dark blue), transcriptional regulatory sequence leader (TRS-L) or body (TRS-B), and polyadenylation (orange). S: Spike; M: Membrane; NC: Nucleocapsid. **B)** Summary of SARS-CoV-2 RNA retrotranspositions in human chromosome X (top) or chromosome 22 (bottom) with flanking host sequences from both sides recovered by Nanopore WGS, published in reference [27].

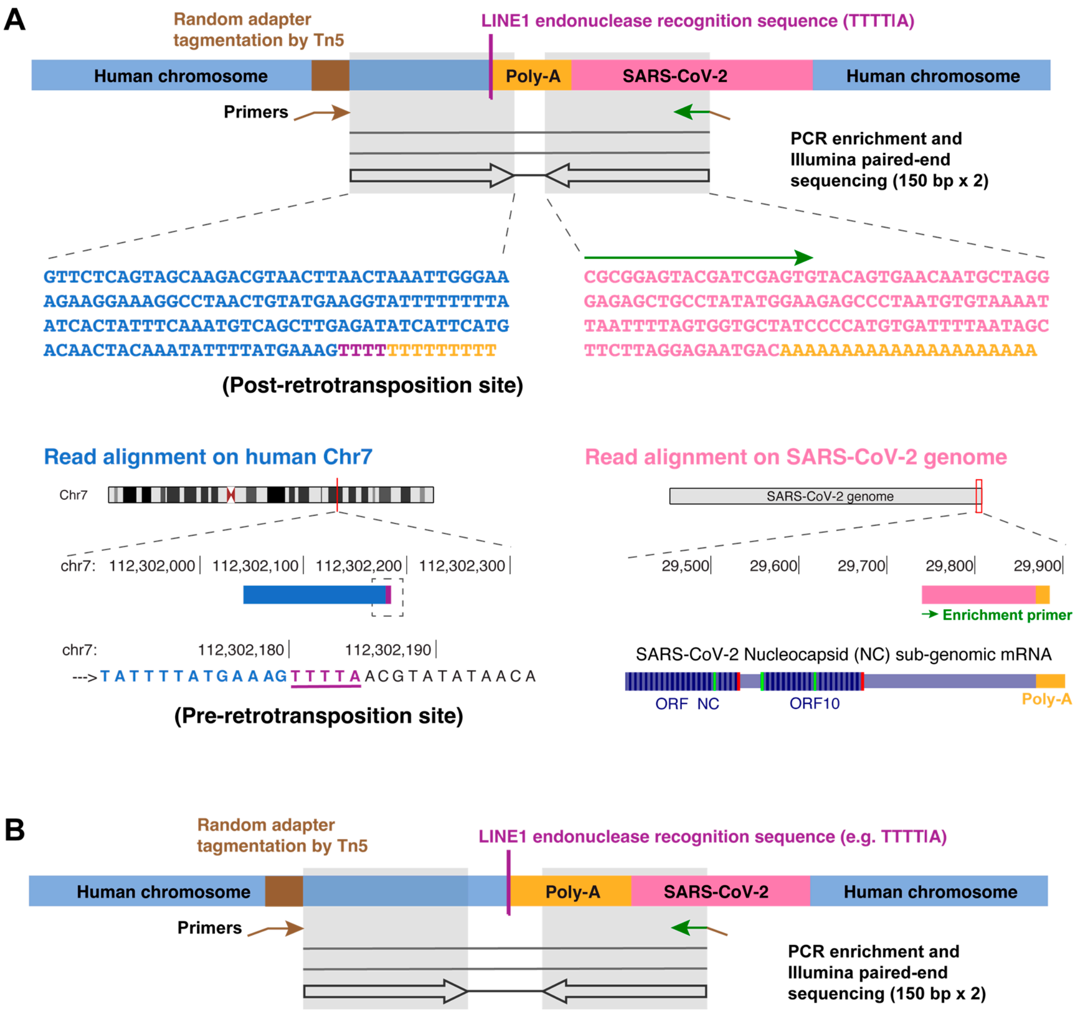

C

Replicate	#1	#2
Integrants with mapped LINE1 EN rec sequence linked to a Poly(A) tract	33	54
Integrants with mapped Poly(A) tract and a putative LINE1 EN rec sequence	645	1056
Total	678	1110

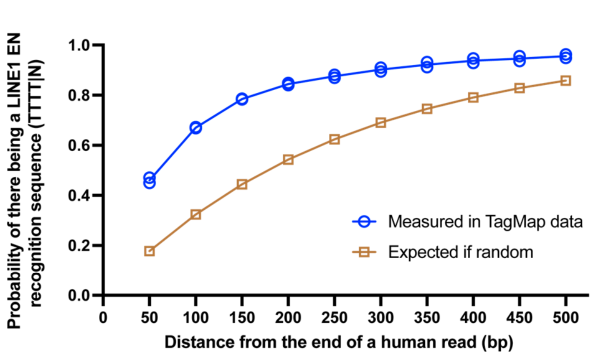

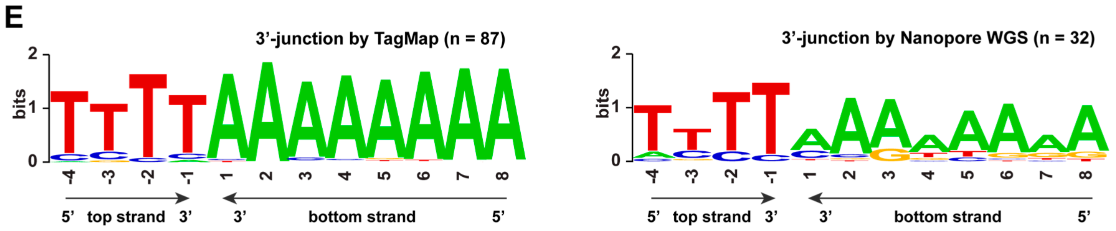

Supplementary Figure S2. TagMap method for enrichment and sequencing of retrotransposition junctions. A) Schematic and an example sequencing read-pair showing the enrichment of one junction of retrotransposed viral cDNA. This method is based on random Tn5 tagmentation on cellular genomic DNA. A primer targeting the inserted adapter sequence (brown arrow) and a primer targeting a SARS-CoV-2 sequence (green arrow) are used to enrich the retrotransposition junction. In this example, the left read mapped to human chromosome 7 (blue) with a LINE1 endonuclease recognition sequence (TTTT/A, purple) linked to a poly-A sequence (poly-A tract, orange). The right read mapped to the 3'-end of a SARS-CoV-2 RNA sequence (pink) starting from the enrichment primer sequence (green arrow) and ending with a poly-A sequence derived from the 3'-end viral RNA poly-A (poly-A tract, orange). **B)** Schematic showing the enrichment of one junction of retrotranspositions by a primer (brown) targeting randomly genomic-inserted adaptor and a viral primer (green arrow) targeting the 3'-end of SARS-CoV-2 RNA sequence. In this case, the left read mapped to a human chromosome. The right read mapped to the 3'-end of a SARS-CoV-2 RNA sequence starting from the enrichment primer sequence (green arrow) and ending with a poly-A tract (orange). LINE1 endonuclease recognition sequence is not directly mapped in the read pair due to read length limitation. **C)** Summary of SARS-CoV-2 RNA retrotranspositions detected by TagMap in LINE1-overexpressing 293T cells. All detected retrotranspositions are evidenced by Illumina read-pairs mapped to human sequences and viral sequences with poly-A tract, with or without a LINE1 endonuclease recognition sequence mapped in the reads. **D)** Probability of seeing a LINE1 recognition sequence motif (TTTT) from the mapped human sequence in certain distance intervals (bp), showing measurements in the TagMap data and the expected probability if this motif (TTTT) appears randomly in a given DNA sequence. **E)** Sequence logos showing patterns of retrotransposition junctions with 3'-flanking host sequence identified by TagMap (left, 87 events from two biological replicates) or Nanopore WGS (right, raw data published in reference [27]) in LINE1 overexpressing 293T cells. Logos were generated using WebLogo (https://weblogo.berkeley.edu/logo.cgi) (see Materials and Methods), covering four nucleotides on the DNA "top strand" upstream of the LINE1 endonuclease cleavage site (positions -4 to -1) and eight nucleotides on the DNA "bottom strand" downstream of the LINE1 endonuclease cleavage site (positions 1 to 8).

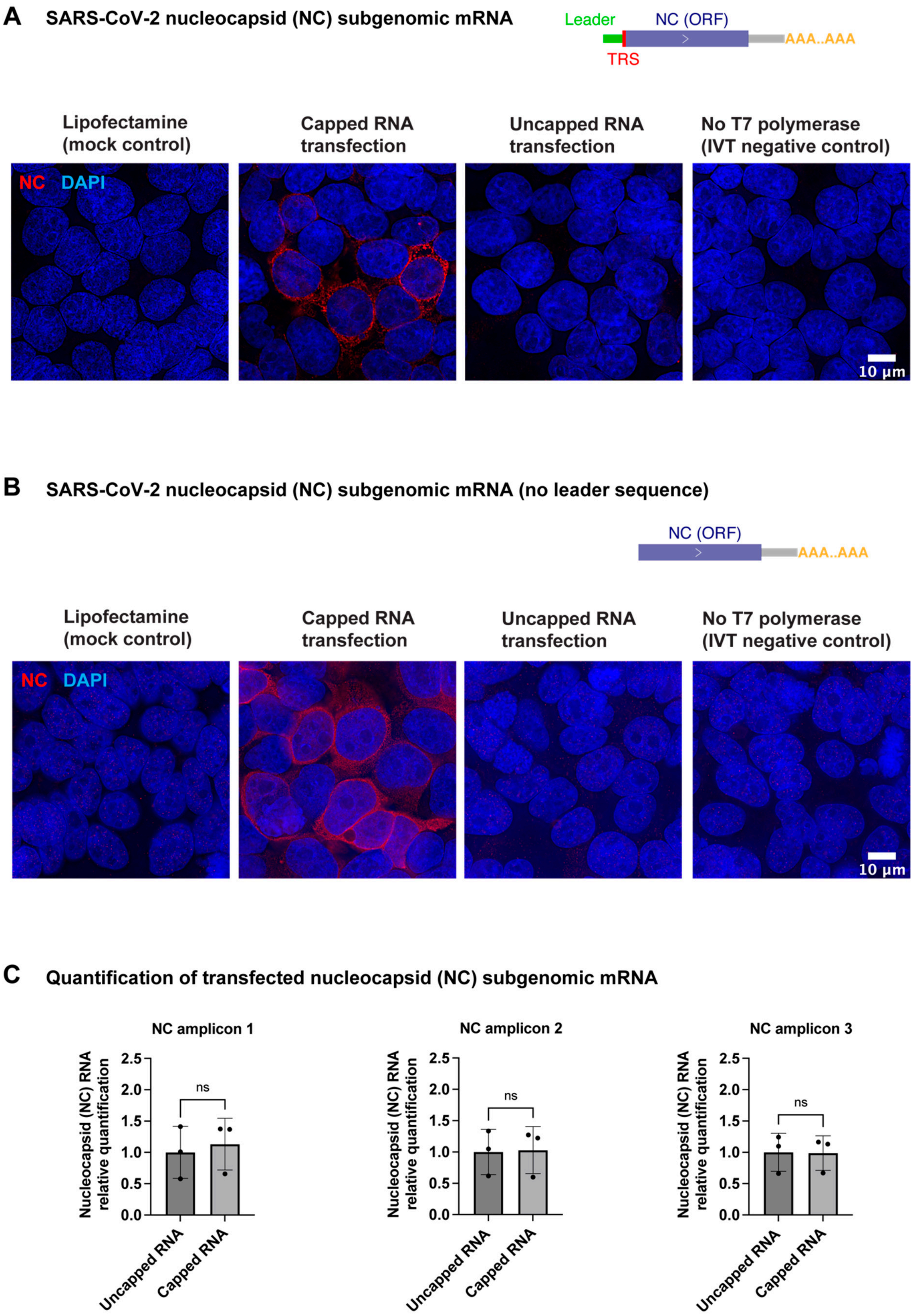
A
SARS-CoV-2 nucleocapsid (NC) subgenomic mRNA
Leader
NC (ORF)
TRS
AAA..AAA
Lipofectamine (mock control)
Capped RNA transfection
Uncapped RNA transfection
No T7 polymerase (IVT negative control)
NC
DAPI
10 μm
B
SARS-CoV-2 nucleocapsid (NC) subgenomic mRNA (no leader sequence)
NC (ORF)
AAA..AAA
Lipofectamine (mock control)
Capped RNA transfection
Uncapped RNA transfection
No T7 polymerase (IVT negative control)
NC
DAPI
10 μm
C
Quantification of transfected nucleocapsid (NC) subgenomic mRNA
NC amplicon 1
NC amplicon 2
NC amplicon 3
Nucleocapsid (NC) RNA relative quantification
2.5
2.0
1.5
1.0
0.5
0.0
ns
Uncapped RNA
Capped RNA

Supplementary Figure S3. SARS-CoV-2 nucleocapsid mRNA expression and quantification in transfected cells. A) Immunofluorescent staining of nucleocapsid (red) and DNA DAPI staining (blue) in 293T cells transfected by mock, SARS-CoV-2 nucleocapsid mRNA with 5'-cap, SARS-CoV-2 nucleocapsid mRNA without 5'-cap, or an *in vitro* transcription negative control eluent (no T7 polymerase). **B)** Immunofluorescent staining of nucleocapsid (red) and DNA DAPI staining (blue) in 293T cells transfected by mock, SARS-CoV-2 nucleocapsid mRNA (no leader sequence, with 5'-cap), SARS-CoV-2 nucleocapsid mRNA (no leader sequence, without 5'-cap), or an *in vitro* transcription negative control eluent (no T7 polymerase). Cells were transfected by RNA (0.5µg RNA per 1mL cell culture medium) for 24 hours. For IVT negative control, a volume of eluent equivalent to the RNA samples was used for transfection. **C)** Relative quantification of transfected uncapped versus 5'-capped SARS-CoV-2 nucleocapsid mRNA (with leader) in 293T cells by RT-qPCR. Cells were transfected by RNA (0.5µg RNA per 1mL cell culture medium) for 24 hours. n = 3 independent experiments (biological replicates). Data are mean ± standard deviation (SD). ns: not significant; $p = 0.714$ (NC amplicon 1), $p = 0.924$ (NC amplicon 2), $p = 0.961$ (NC amplicon 3); unpaired two-tailed t-test to compare uncapped versus capped RNA relative quantification.

A SARS-CoV-2 nucleocapsid (NC) subgenomic mRNA

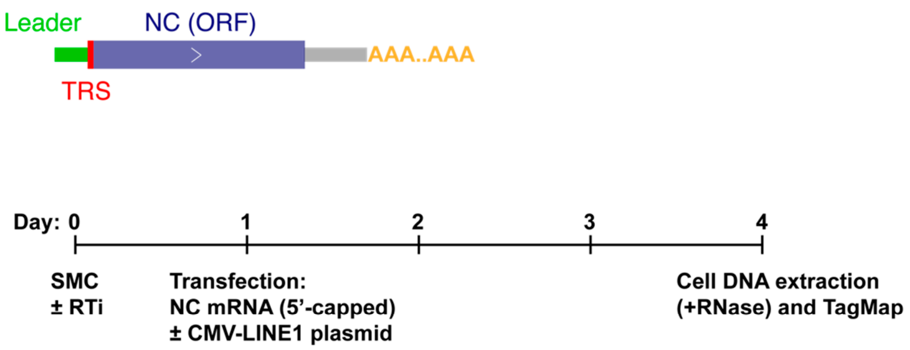

B

Cell	Retrotranspositions detected by TagMap	
	- RTi	+ RTi
SMC	0	-
SMC (+ CMV-LINE1 expression plasmid)	8	3

Supplementary Figure S4. No retrotransposition events detected by TagMap in vascular smooth muscle cells transfected by viral nucleocapsid (NC) mRNA alone. A) Schematics showing the SARS-CoV-2 NC mRNA architecture and the experimental workflow. **B)** Retrotranspositions detected by TagMap in vascular smooth muscle cells transfected with SARS-CoV-2 NC subgenomic mRNA, with or without LINE1 overexpression. Vascular smooth muscle cells were transfected by the NC mRNA with a concentration of 1µg RNA per 1.2mL cell culture medium. For LINE1 overexpression, a CMV-LINE1 plasmid was co-transfected with the NC mRNA, with a concentration of 1µg plasmid per 1.2mL cell culture medium. One set of LINE1-overexpressing cells was treated with reverse transcriptase inhibitors (RTi, 100µM AZT and 10 µM ABC) 16 hours before RNA and plasmid transfection. New RTi were added at the day of and the day after transfection.

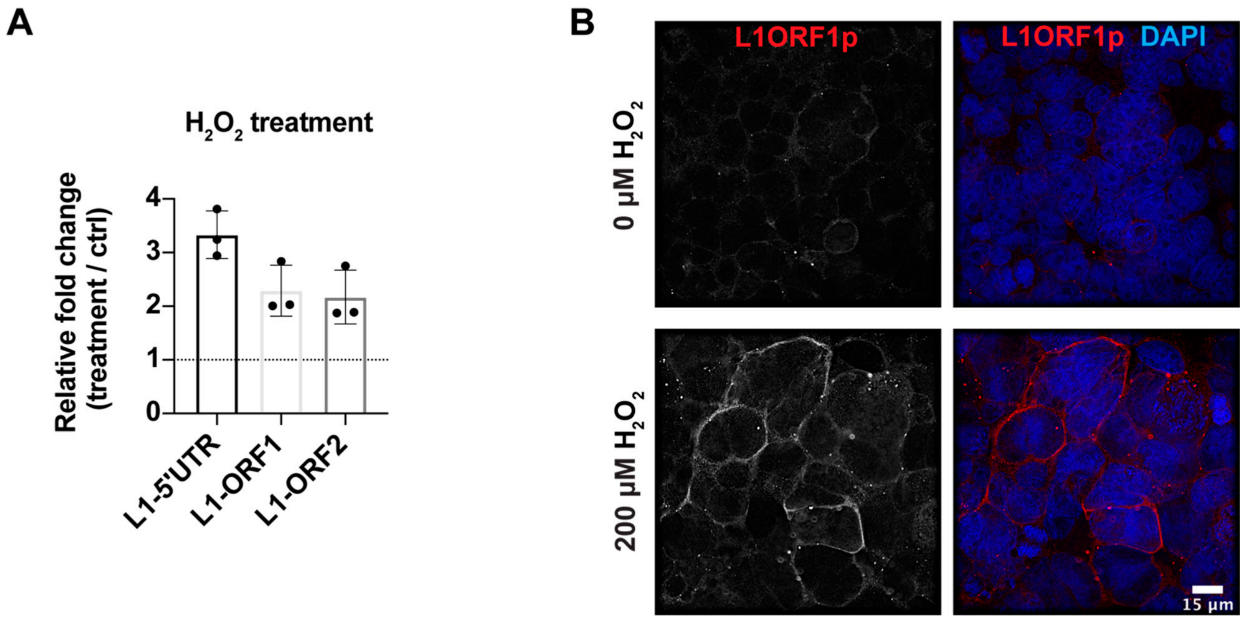

Supplementary Figure S5. Oxygen stress can induce endogenous LINE1 expression in cultured 293T cells. A) LINE1 expression fold-change detected by RT-qPCR in 293T cells treated by 200µM hydrogen peroxide for 2 hours followed by 24 hours of culturing. RT-qPCR was performed using L1HS/L1PA(2-6) specific primers targeting LINE1 5'-UTR or ORF1, and L1HS specific primers targeting LINE1 ORF2, on purified cellular poly-A RNA (method and primer sequences following a previous publication [2], see Materials and Methods). n = 3 independent experiments (biological replicates). Data are mean ± standard deviation (SD). One-tailed t-test for LINE1 mRNA upregulation in hydrogen peroxide treated cells: $p = 0.112$ (L1-5'UTR), $p = 0.154$ (L1-ORF1), $p = 0.087$ (L1-ORF2). **B)** Immunofluorescent staining of L1ORF1p (red) and merged channels with DAPI staining (blue) in PBS or hydrogen peroxide treated 293T cells. Cells were treated by 200µM hydrogen peroxide for 2 hours followed by 3 days of culturing.
